# Differences by ethnicity in the association between unpaid caring and health trajectories over 10 years in the UK Household Longitudinal Study

**DOI:** 10.1136/jech-2024-222633

**Published:** 2024-09-30

**Authors:** Whitney Wells, Baowen Xue, Rebecca Lacey, Anne McMunn

**Affiliations:** 1 Department of Epidemiology and Biostatistics, University of California San Francisco, San Francisco, California, USA; 2 Research Department of Epidemiology & Public Health, University College London, London, UK; 3 School of Health and Psychological Sciences, City St George's, University of London, London, UK

**Keywords:** COHORT STUDIES, ETHNIC GROUPS, Health inequalities, LONGITUDINAL STUDIES, AGING

## Abstract

**Background:**

Unpaid carers deliver critical social care. We aimed to examine differences by ethnicity in (1) profiles of unpaid caring and (2) associations between caring and physical and mental health trajectories.

**Methods:**

We used 10 waves of data from 47 015 participants from the UK Household Longitudinal Study (2009–2020). Our outcomes were 12-item Short Form Health Survey physical and mental component scores. We performed bivariate comparison of profiles of caring by ethnicity. We used multilevel linear mixed effects models to estimate associations between caring and health trajectories and assess for heterogeneity by ethnicity.

**Results:**

We found that caring profiles differed by ethnicity. The proportion caring for someone within their household ranged from 39.7% of White carers to 70.1% of Pakistani and 74.8% of Bangladeshi carers. The proportion providing 20+ hours/week of care ranged from 26.9% of White carers to 40.6% of Pakistani and 43.3% of Black African carers. Ethnicity moderated associations between caring and physical but not mental health trajectories (test for interaction: p=0.038, p=0.75). Carers showed worse physical health compared with non-carers among Black African (−1.93; −3.52, –0.34), Bangladeshi (−2.01; −3.25, –0.78), Indian (−1.30; −2.33, –0.27) and Pakistani carers (−1.16; −2.25, –0.08); Bangladeshi carers’ trajectories converged with non-carers over time (0.24; −0.02, 0.51). White carers showed better baseline physical health than non-carers (0.35; 0.10, 0.60), followed by worsening trajectories versus non-carers (−0.14; −0.18, –0.10).

**Conclusions:**

There are differences by ethnicity in profiles of caring and associations between caring and physical health trajectories. Future research should account for ethnicity to ensure applicability across groups.

**WHAT IS ALREADY KNOWN ON THIS TOPIC**

- Unpaid carers provide the majority of care in the UK and caring has been shown to impact carers’ health.
- While some research has suggested differences in the prevalence and effect of caring by ethnicity in the UK, in-depth quantitative evidence is lacking.

**WHAT THIS STUDY ADDS**

- Exposure to caring—especially intensive caring—differs by ethnicity in the UK.
- Ethnicity moderates the association between caring and physical health trajectories.

**HOW THIS STUDY MIGHT AFFECT RESEARCH, PRACTICE OR POLICY**

- The UK government should prioritise national policy to support carers as rising needs for unpaid caring may exacerbate inequities in the burden of unpaid caring in the UK.

## Introduction

 Unpaid carers provide the majority of care in the UK, filling a critical role in the health and social service sectors. The work of unpaid carers (who take care of family or friends needing support due to illness, disability or old age) has been valued at £132 billion per year and rising.^1^ Roughly 5 million people in England and Wales are unpaid carers, with 1.5 million caring >50 hours/week.^2^ The need for unpaid carers is projected to rise by 63% from 2015 to 2035.^3^

Evidence suggests caring impacts the health of carers; carers show worse self-reported health,^4^ higher cholesterol^5^ and higher adiposity in women.^6^ Women providing long-term or intermittent care show slightly elevated psychological distress versus non-carers from initiation of care,^7^ and carers show increased psychological distress at transition to caring, especially caring more than 20 hours/week or for someone in the household.^8^ On the other hand, some research found elderly female carers have a lower risk of mortality than non-carers, suggesting a ‘healthy carer’ hypothesis of healthier people being more able to take on caring.^9^

### Heterogeneity in caring by ethnicity

Evidence has suggested heterogeneity in the prevalence and effect of caring by ethnicity.^10^,^12^ Structural racism and the social construction of ethnicity are tied to unequal hierarchical social positions by race/ethnicity, producing inequities in the distribution of fundamental causes of health, that is, access to flexible resources and social determinants of health.^13 14^ Research has also highlighted differential effects of these resources as social factors such as structural racism diminish the potential for minoritised individuals to leverage flexible resources (leading to ‘diminished returns’).^15^,^18^ Inequities by ethnicity in access to—and ability to benefit from—flexible resources and social determinants of health may influence the health of carers and care recipients, and likelihood of providing unpaid care and intensity of exposure to caring.^19^

Evidence on caring by ethnicity in the UK is limited. The 2021 census in England and Wales shows differences in the age-standardised prevalence of unpaid caring by ethnicity, ranging from roughly 6% (among Black African individuals) to 10% (among White British individuals).^20^ Research has found ethnic minority carers more likely to care for someone with mental health problems, more young adult carers who are Indian, Pakistani, or Bangladeshi,^11^ and higher depression among South Asian than White British carers, although these all used cross-sectional data.^10^ 2014 research found higher anxiety and depression among British Indian than White British carers, although this was based on small samples.^12^ Caring charity and advocacy organisations highlight that ethnic minority carers are under-represented in research on caring impacts and intervention evaluation.^21^ We hope to contribute to the representation of diverse experiences and the impact of caring by ethnicity.

To understand potential health disparities by ethnicity, it is important to consider effect modification and potential differences in prevalence and intensity of exposure.^22^ We, therefore, sought to examine potential differences in both caring profiles as well as associations between caring and health in the UK. Objective 1: examine whether caring status and care characteristics differ by ethnicity. Objective 2: examine whether associations between caring and 12-item Short Form Health Survey (SF-12) mental and physical health trajectories differ by ethnicity.

## Materials and methods

### Study population

We used the UK Household Longitudinal Study (UKHLS), a nationally representative longitudinal study of roughly 40 000 households with participants interviewed roughly annually.^23^ More details are found in the online supplemental file and reported elsewhere.^24^ In wave 1 (W1), the sample was supplemented by an ethnic minority boost of over 4000 households. Our analysis includes data from W1 (fielded December 2008 to March 2011) to W10 (fielded December 2017 to May 2020).

**Figure 1:**
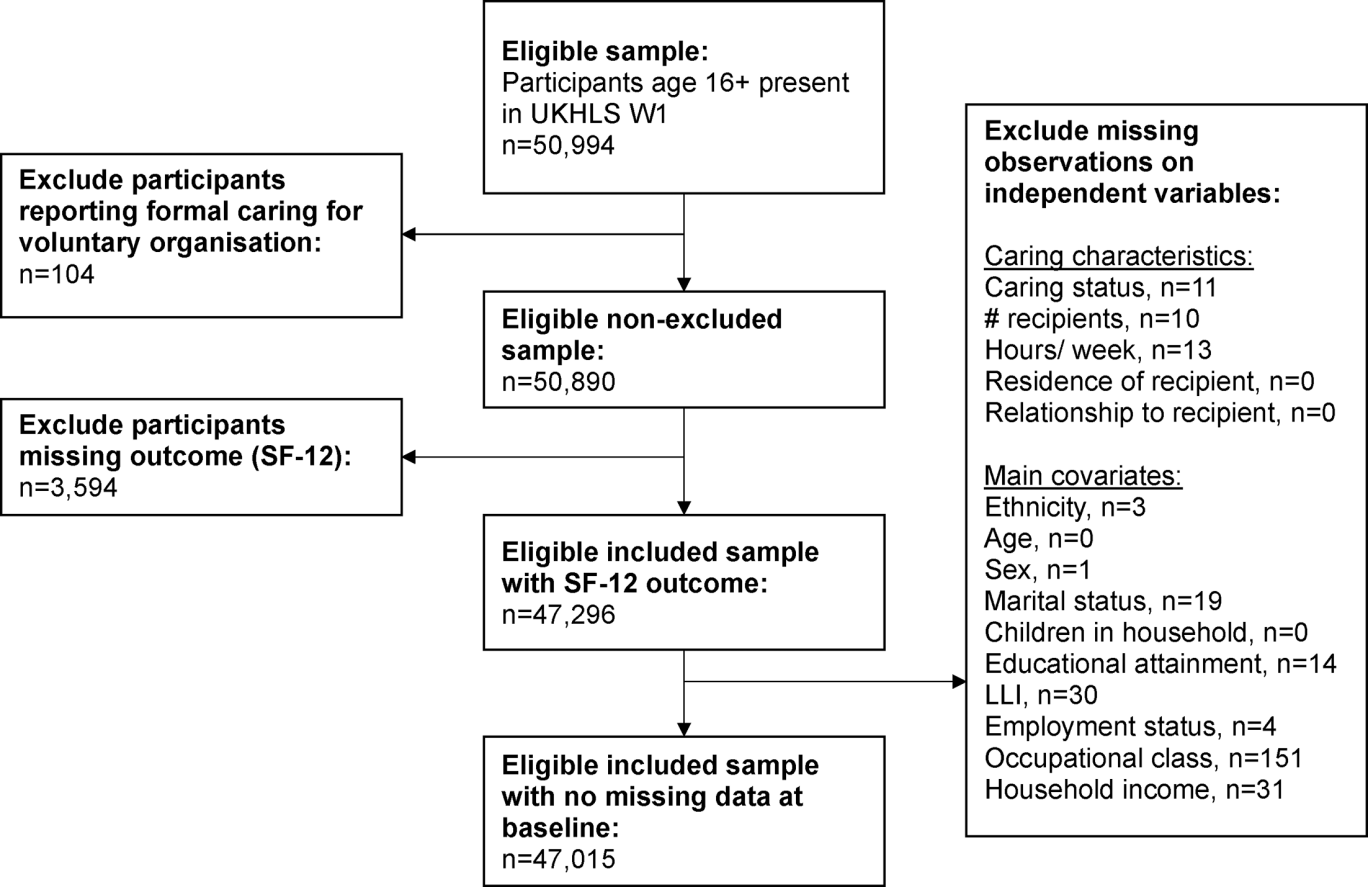
Flow diagram of study sample inclusion. LLI, limiting long-standing illness; SF-12, 12-item Short Form Health Survey; UKHLS, UK Household Longitudinal Study; W1, wave 1.

All participants aged 16+ in W1 were eligible for inclusion. We excluded those caring for clients of voluntary organisation, given our focus was the role of informal unpaid caring outside of any formal caring arrangement that could include volunteer organisations (detail in online supplemental file). We excluded those missing W1 exposure, outcome or covariates (figure 1). Final sample was 47 015 (92.2% of initial sample); online supplemental eTable 1 shows the contributing sample size for each wave.

### Outcomes

The SF-12 is a standard measure of self-reported health, measured every wave in UKHLS, providing a physical component summary (PCS) and mental component summary scale score, where higher scores represent better health (detail in online supplemental file).^25^

### Exposure

Caring status was measured each wave using two questions: ‘*Is there anyone living with you who is sick, disabled or elderly whom you look after or give special help to (for example, a sick, disabled or elderly relative, husband, wife or friend etc)?’* and ‘*Do you provide some regular service or help for any sick, disabled or elderly person not living with you?’*. Participants were coded as carers if they responded ‘Yes’ to either question. Our main exposure was defined as W1 caring status.

Four additional care characteristics were included: hours per week, residence of recipient(s), number of recipient(s) and relationship to recipient(s) (detail in online supplemental file). We were unable to account for care recipient condition or age of recipient.

### Covariates

UKHLS includes self-identification of ethnicity using the 2011 census question.^24^ The ethnic minority boost sample aimed to provide ≥1000 adult participants in five groups: Black African, Bangladeshi, Black Caribbean, Indian and Pakistani.^24^ We created the following categories: Black African, Bangladeshi, Black Caribbean, Indian, Pakistani, White British and other. We reported results by ethnicity in alphabetical order (detail in online supplemental file).

We adjusted for the following potential confounders using W1 data to avoid adjusting for postexposure variables: age, sex, marital status, number of own children in the household, highest educational attainment, employment status, occupational class, net monthly equivalised household income and baseline limiting long-standing illness (LLI) (excluded from models with physical health outcome) (detail in online supplemental file). In models stratified by ethnicity, we additionally controlled for nativity (whether an individual was born inside or outside the UK) based on existing evidence regarding the intersecting roles of socially constructed ethnicity and nativity.^26^

### Statistical methods

Statistical analyses were performed in Stata V.17. To assess potential bias from missing data, we compared our analytical sample to sample excluded for missingness. We used χ^2^ tests for bivariate associations between baseline participant characteristics and caring. We included UKHLS survey weights at baseline to correct for unequal selection probability and non-response (detail in online supplemental file).

In objective 1, we examined differences in caring profiles at W1 (2009–2011) by ethnicity. We used χ^2^ tests for bivariate associations between ethnicity and the four baseline care characteristics described above.

In objective 2, we examined whether there were differences in the association between W1 caring and SF-12 trajectories from W1 to W10 (2009–2020). We estimated the association between caring and health trajectories using multilevel linear regression (growth curve models) to account for correlation between repeated measures within an individual (‘mixed’ package in Stata). Models included wave, wave squared, interaction between W1 caring and wave, and random slope for wave and wave squared. All included a quadratic wave term given improved model fit. We graphed health trajectories based on estimated average marginal effects (detail in online supplemental file). Model 1 adjusted for baseline age and sex, and model 2 additionally adjusted for remaining covariates.

We assessed effect modification by ethnicity via a triple interaction term between ethnicity, W1 caring and wave. Where we found evidence for differences, we stratified by ethnicity. Our main results present the stratified adjusted model. Given some of the proposed confounders may actually serve as mediators (eg, education, employment, occupational class, income) for the effect modification by ethnicity, we also conducted stratified analysis without adjusting for these factors in the supplement. Comparing these results is valuable; while the adjusted model may block some of the effect modifier’s pathway, the crude model may leave the main exposure confounded. The true estimate may be hypothesised to lie between these estimates. Reassuringly, the results from the two specifications were very similar.

## Results

### Sample characteristics

Table 1 summarises the sample characteristics at baseline. 16.4% were carers. Distribution of ethnic groups was: 1.3% Black African, 0.5% Bangladeshi, 0.9% Black Caribbean, 2.3% Indian, 1.1% Pakistani and 86.5% White. Carers were more likely to be Bangladeshi, White and Pakistani (difference between Bangladeshi and Pakistani not visible due to rounding). Carers had higher mean age and were more likely to be female, married, have LLI and be retired or looking after the home/family. They were less likely to have a degree, be employed, or in management/professional occupational class, and had lower household income.

**Table 1:** Sample characteristics by caring status at wave 1

	Carer (n=7798)	Not carer (n=39 217)	Total (n=47 015)		P value for % difference
	%/mean (SE)	%/mean (SE)	%/mean (SE)	N*	
Ethnicity					<0.001
Black African	0.4	1.4	1.3	1391	
Bangladeshi	0.5	0.5	0.5	1104	
Black Caribbean	0.8	0.9	0.9	1109	
Indian	1.7	2.4	2.3	1868	
Pakistani	1.1	1.1	1.1	1407	
White	91.1	85.6	86.5	35 484	
Other	4.3	8.0	7.4	4652	
Age (years)	50.81 (0.26)	45.60 (0.15)	46.45 (0.14)	47 015	<0.001
Sex					<0.001
Male	42.8	50.0	48.8	20 699	
Female	57.2	50.0	51.2	26 316	
Marital status					<0.001
Married/civil partner	60.5	47.4	49.6	23 871	
Living as couple	10.1	12.9	12.4	5324	
Widowed	3.9	7.3	6.7	2832	
Separated/divorced	8.3	7.5	7.6	4178	
Never married	17.3	24.9	23.6	10 810	
Number of own children <16 in household					<0.001
0	74.8	72.8	73.1	33 044	
1	11.9	12.5	12.4	6188	
2	8.7	10.7	10.3	5283	
3+	4.6	4.1	4.2	2500	
Highest qualification					<0.001
Degree	16.6	21.7	20.8	10 172	
Other higher education	12.3	11.0	11.2	5256	
A-Level/equivalent	18.8	19.9	19.7	8824	
GCSE/equivalent	21.8	20.8	21.0	9691	
Other qualification	13.4	10.0	10.5	4866	
No qualification	17.0	16.6	16.7	8206	
LLI					<0.001
No	69.0	76.4	75.2	35 460	
Yes	31.0	23.6	24.8	11 555	
Employment status					<0.001
Employed	50.9	56.7	55.8	25 143	
Unemployed	6.5	5.9	6.0	3146	
Retired	26.0	21.1	21.9	9642	
Family/home care	8.9	4.8	5.5	3501	
Student/training	3.1	7.5	6.8	3463	
LT sick/disabled	3.4	3.4	3.4	1789	
Other	1.2	0.6	0.7	331	
Occupational class					<0.001
Management/professional	19.7	24.0	23.3	10 535	
Intermediate	13.2	13.7	13.6	6137	
Routine	19.1	21.2	20.8	9300	
Not employed	48.1	41.2	42.3	21 043	
Net equivalised monthly household income (£)	1481.26 (15.70)	1522.41 (9.45)	1515.65 (8.68)	47 015	0.015

Source: UK Household Longitudinal Study, Wave 1.

* Sample size unweighted, percentage weighted.

GCSEGeneral Certificate of Secondary EducationLLIlimiting long-standing illnessLTlong-termNsample size

Online supplemental eTable 2 compares the analytical sample with cases excluded due to missingness. The included sample were slightly more likely to be carers, White, female, aged 50+, separated/divorced, have children, higher education, LLI, not be employed, be in the lowest income tertile and have lower mean SF-12 PCS. However, our final sample included 92.2% of our initial eligible sample, and potential bias due to complete case analysis has been found to be relatively low in analyses with missingness <10% and large sample sizes.^27^

### Objective 1: caring characteristics by ethnicity

The proportion of carers by ethnicity was: Bangladeshi (17.6%), White (17.3%), Pakistani (16.4%), Black Caribbean (15.6%), Indian (11.8%) and Black African (5.6%) (online supplemental eTable 3). Table 2 shows the care characteristics by ethnicity. Bangladeshi (74.8%) and Pakistani (70.1%) carers were most likely to be coresident caring (inside the home), while White carers were least likely (39.7%). Pakistani carers were most likely to be caring for 2+ care recipients (22.9%), while Black African carers were least likely (14.1%). Black African (43.3%) and Pakistani (40.6%) carers were most likely to be caring for 20+ hours/week, while White carers were least likely (26.9%). Caring for a child was most prevalent in Black African (23.8%), Bangladeshi (14.2%) and Black Caribbean (19.2%) carers. Caring for a parent was most prevalent in Indian (54.7%) and Pakistani (57.0%) and least prevalent in Black African (24.1%) carers. Caring for a partner was most prevalent among Black African (19.1%) and White (20.9%) carers.

**Table 2:** Care characteristics by ethnicity among carers at wave 1

	Black African (n=80)	Bangladeshi (n=194)	Black Caribbean (n=174)	Indian (n=230)	Pakistani (n=250)	White (n=6383)	Other (n=487)	Total (n=7798)		P value for % difference
	%/mean (SE)	%/mean (SE)	%/mean (SE)	%/mean (SE)	%/mean (SE)	%/mean (SE)	%/mean (SE)	%/mean (SE)	N*	
**Location of recipient(s)**										<0.001
In home only	56.2	70.2	45.7	56.7	59.9	34.5	41.0	35.9	2832	
Outside home only	41.7	25.2	48.9	39.9	29.9	60.2	54.6	59.0	4555	
Both	2.1	4.6	5.5	3.3	10.2	5.2	4.4	5.2	411	
**Number ofrecipients**										0.66
1	85.9	84.0	83.2	81.9	77.1	79.5	81.1	79.7	6216	
2+	14.1	16.0	16.8	18.1	22.9	20.5	18.9	20.3	1582	
**Hours/week caring**										0.002
0–4	15.3	29.1	34.7	35.3	18.9	37.6	36.6	37.1	2766	
5–9	20.5	13.2	16.2	13.2	16.7	18.5	16.3	18.2	1418	
10–19	15.2	16.0	10.6	10.4	19.2	12.0	12.4	12.1	989	
20+	43.3	36.0	34.1	35.1	40.6	26.9	29.3	27.5	2252	
Other	5.7	5.7	4.5	5.9	4.5	5.0	5.5	5.1	373	
**Relationship to recipient (% yes)**										
Child	23.8	14.2	19.2	9.3	9.9	9.3	12.2	9.6	805	0.002
Parent	24.1	47.6	43.8	54.7	57.0	46.5	41.8	46.4	3623	0.001
Partner	19.1	16.2	12.0	14.0	17.9	20.9	18.6	20.6	1586	0.048
Other	37.4	28.1	31.4	27.5	23.4	31.1	35.0	31.1	2392	0.21

Source: UK Household Longitudinal Study, Wave 1.

* Sample size unweighted, percentage weighted.

Nsample size

### Objective 2: caring and physical and mental health trajectories by ethnicity

**Figure 2:**
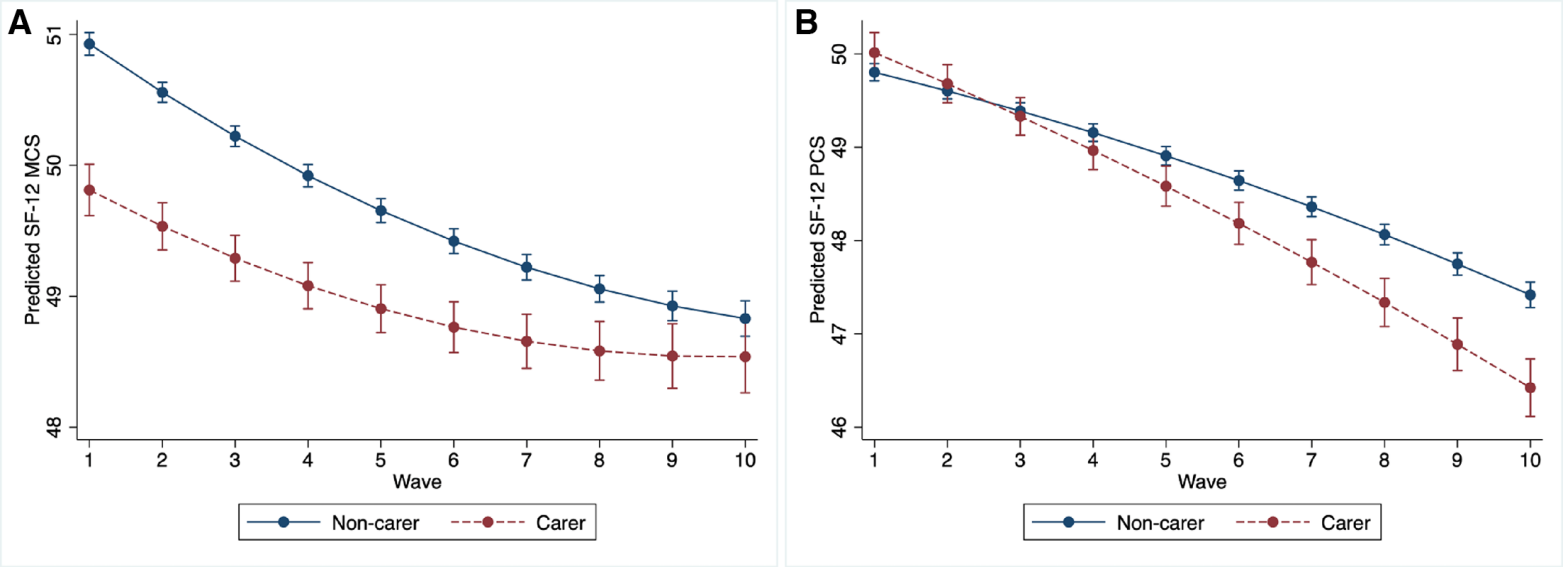
Predicted SF-12 MCS (**A**) and SF-12 PCS (**B**) waves 1–10. Source: UK Household Longitudinal Study, Waves 1–10. Weighted using survey weight at baseline. Model adjusted for baseline age, sex, marital status, number of own children under 16 in household, highest educational qualification, LLI (for MCS), employment status, occupational class and net equivalised monthly household income. Model includes linear and quadratic term for wave. LLI, limiting long-standing illness; MCS, mental component summary; PCS, physical component summary; SF-12, 12-item Short Form Health Survey.

We found evidence for an overall association between caring and mental health trajectories, with carers showing worse mental health at baseline (change in SF-12: −1.11; 95% CI −1.33, –0.90), but mental health trajectories of carers converged with those of non-carers over time (0.09; 0.06, 0.13) (figure 2, online supplemental eTable 4). However, we did not find evidence of heterogeneity in this association by ethnicity based on the interaction term for caring, ethnicity and wave (online supplemental eTable 5; test for interaction: p=0.75).

**Figure 3:**
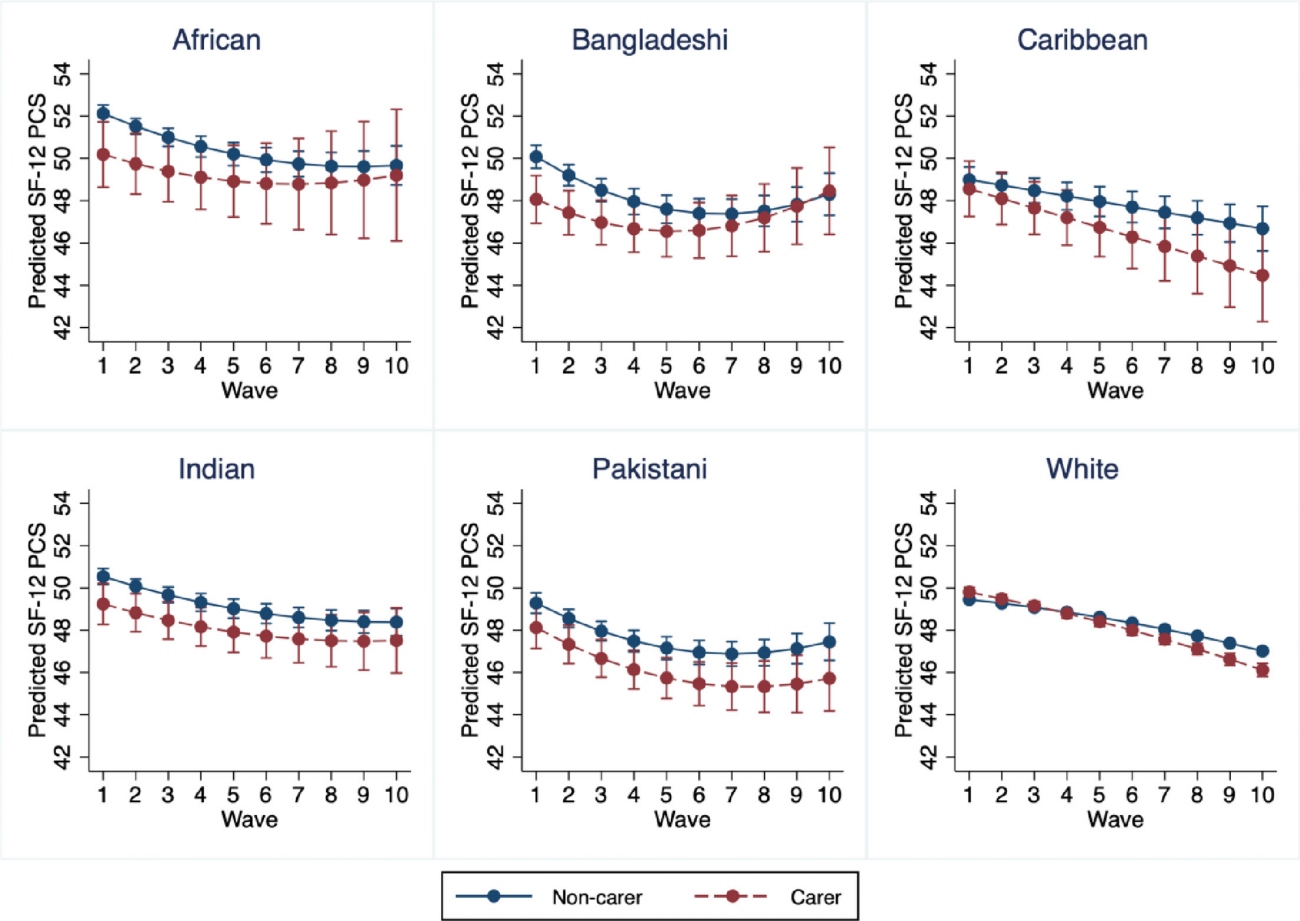
Predicted SF-12 PCS waves 1–10 for UKHLS adults by ethnicity. Source: UK Household Longitudinal Study, Waves 1–10. Model adjusted for baseline age, sex, marital status, number of own children under 16 in household, highest educational qualification, employment status, occupational class, net equivalised monthly household income and nativity. Model includes linear and quadratic term for wave. PCS, physical component summary; SF-12, 12-item Short Form Health Survey; UKHLS, UK Household Longitudinal Study.

We found evidence for an association between caring and physical health trajectories, with carers showing better physical health at baseline (0.21; −0.03, 0.45), followed by a physical health trajectory that worsened more rapidly over time than for non-carers (−0.13; −0.17, –0.10) (figure 2, online supplemental eTable 4). We found evidence of heterogeneity in this association by ethnicity based on the interaction term for caring, ethnicity and wave (online supplemental eTable 5; test for interaction: p=0.038). Figure 3 shows conditional growth curves stratified by ethnicity (detailed results: online supplemental eTable 6; growth curves for the crude model: online supplemental eFigure 1). These results should be interpreted cautiously given wide and overlapping CIs.

Among Bangladeshi individuals, caring was associated with slightly worse baseline physical health (−2.01; −3.25, –0.78), and carers’ physical health trajectories converged with non-carers over time (0.24; −0.02, 0.51). Among three of the ethnic groups, we found slightly worse baseline physical health followed by similar physical health trajectories over time among carers versus non-carers. These were: Black African (baseline: −1.93; −3.52, –0.34, change each wave: 0.16; −0.22, 0.55), Indian (baseline: −1.30; −2.33, –0.27, change each wave: 0.05; −0.15, 0.24) and Pakistani (baseline: −1.16; −2.25, –0.08, change each wave: −0.06; −0.27, 0.15). Among White individuals, caring was associated with slightly better baseline physical health (0.35; 0.10, 0.60), followed by a physical health trajectory that worsened more rapidly over time than for non-carers (−0.14; −0.18, –0.10). Among Black Caribbean individuals, we did not find strong evidence for a difference in baseline physical health (−0.43; −1.86, 0.99), or differences in physical health changes each wave between carers and non-carers (0.20; −0.47, 0.07). However, CIs are wide and overlapping. Some stratified graphs show upward trend in physical health at later waves, possibly due to attrition of individuals with lower physical health.

## Discussion

We found that profiles of caring and intensity of caring differed by ethnicity. Caring prevalence was relatively high among Bangladeshi, White and Pakistani individuals. Coresident caring was most prevalent among Bangladeshi and Pakistani carers, caring for 2+ recipients was most prevalent among Pakistani carers and caring 20+ hours/week was most prevalent among Pakistani and Black African carers. Exposure to more intense caring may influence how caring impacts physical health over time.

We also found heterogeneity by ethnicity in associations between caring and physical but not mental health trajectories. Although in pooled analysis carers showed better baseline physical health than non-carers, in stratified analysis this association was only evidenced among White individuals, and carers showed worse baseline physical health than non-carers among Black African, Bangladeshi, Indian and Pakistani individuals. Although in pooled analysis carers showed a greater decline in physical health compared with non-carers, in stratified analysis this was only evidenced among White individuals, and we saw the opposite effect among Bangladeshi carers versus non-carers.

While our research did not examine potential pathways for the observed heterogeneity in profiles of caring, existing research notes some potential pathways. The demographic make-up of ethnic groups in the UK has been differentially shaped by historical colonialism and immigration policies, leading to differences in migration histories, age profiles, health and family patterns by ethnicity,^28^ which may influence profiles of caring (eg, relationship to care recipient or intensity of care). In qualitative research, South Asian and Black Caribbean unpaid carers reported greater familial expectation of caring,^29^ which may influence intensity of caring (eg, coresident care or hours per week). Ethnic minority carers are more likely to face barriers to accessing caring services,^10 11^ language barriers^30^ and lower financial ability to hire caring support if needed, linked to the historical and contemporary effects of structural racism.^11^ In our research, we found higher exposure to intense caring among Bangladeshi and Pakistani carers, which could be linked to the factors discussed above.

In addition, community-based organisations have highlighted other ways structural racism has contributed to potential inequities, as ethnic minority carers face higher risk of marginalisation from health and welfare systems, and are more likely to face poverty, unemployment, area deprivation, social exclusion and institutional racism.^31^ Beyond differential intensity of exposure to caring, these factors could separately influence the physical health impact of providing care by influencing the burden placed on unpaid carers and whether individuals in poor physical health themselves still take on care. However, more research into potential mechanisms for differential associations between caring and physical health is needed.

In contrast with some (limited) UK cross-sectional research,^10 12^ we did not find evidence that ethnicity moderated associations between caring and mental health. There are a few reasons our research may differ: prior research compared carers’ mental health across ethnic groups (rather than comparing whether associations between caring and mental health differed by ethnicity), which is likely to be influenced by existing differences in mental health across ethnic groups that may not be related to caring. Also, we used a population-based (rather than purposive) sample.

There are a few possible interpretations of differences in baseline physical health by ethnicity. This could represent a selection effect of who takes on caring, with White individuals in poor physical health less likely to take on caring than Bangladeshi and Indian individuals in poor health. Alternatively, this could represent either the immediate impact of initiation of care (if W1 carers on average initiated care recently), or the long-term impact of caring (if they have been caring for extended period).

Our research addresses a gap in research on caring by ethnicity using population-based data. UKHLS is a large, nationally representative, longitudinal dataset with detailed data on caring and health status and covariates among both carers and non-carers. We were able to use growth curve modelling, accounting for correlation between repeated measures within individuals. UKHLS’ ethnic minority boost sample allowed us to avoid combining ethnic groups for five boosted groups often previously combined.

There are several limitations. We lack access to caring trajectories prior to W1 so are unable to differentiate between new and ongoing carers and may face residual confounding due to prior caring trajectories. We were unable to accommodate changes in exposure over time; after W1, carers may have maintained, discontinued or intermittently provided care, which could influence the impact of caring.^7 8^ Nonetheless, our approach provides valuable insight on health trajectories after caring at a single time point, akin to a target trial framework examining an intervention irrespective of subsequent exposure. Alternatively, if our exposure of interest were the impact of long-term exposure to caring, our approach would represent intention-to-treat analysis with caring changes after W1 representing misclassification from the W1 definition; this would tend to influence results towards the null under a true effect.^32^ Additionally, there may be differential misclassification of exposure by ethnicity; research highlights ethnic minority carers are less likely to self-identify as carers.^21^ We were unable to explore the full nuance of ethnicity or intersectional identities. Although we separately analysed the boosted groups, we faced small cell sizes and limited power in stratified analysis and other groups could not be individually examined. We were unable to analyse other dimensions of care (carer age, relationship to recipient, recipient condition). There is potential for bias due to missing data, especially due to attrition.

## Conclusions

This research contributes evidence of heterogeneity by ethnicity in profiles of caring and in the association between caring and physical but not mental health trajectories in the UK. We found that pooled associations between caring and physical health are reflected only among White individuals, suggesting that evidence that does not account for ethnicity may fail to represent experiences of carers in other ethnic groups. Our findings highlight the importance of avoiding grouping heterogeneous ethnic groups given noteworthy differences in previously combined subgroups. Finally, this research supports the importance of national policy to support carers, as growing need for unpaid caring^3^ could exacerbate existing inequities in the distribution of unpaid caring across society.

## supplementary material

10.1136/jech-2024-222633 online supplemental file 1

## Data Availability

Data are available in a public, open access repository.
