# Supplementary material for: Differences by ethnicity in the association between unpaid caring and health trajectories over 10 years in the UK Household Longitudinal Study
Source: J Epidemiol Community Health. 2024 Sep 30;79(2):e222633. doi: 10.1136/jech-2024-222633 (PMC11874286; doi:10.1136/jech-2024-222633)
Supplement: online supplemental file 1 [file jech-79-2-s001.pdf]

Online Supplemental Materials

Methods

We followed Strengthening the Reporting of Observational Studies in Epidemiology (STROBE) guidelines for cohort studies.[1]

Study population

UKHLS covers a range of subjects, including health, work, family, and social life. The main questionnaire is completed by everyone in the household aged 16+ face-to-face or online. UKHLS data are accessible via UK Data Service. We excluded data from wave 11 onward due to the impact of the Covid-19 pandemic on our outcomes of interest and on UKHLS fieldwork. Fieldwork for each wave of UKHLS spans a 24-month period that overlaps with previous and/or subsequent waves (i.e. Wave 1 fieldwork was conducted Dec 2008 – Mar 2011, Wave 2 fieldwork was conducted Jan 2010 – Mar 2012); individual participants are interviewed approximately annually.

The relationship to care recipient(s) was captured via the following question *"Who is the person that you look after or help?"* The options were "Parent/parent-in-law," "Grandparent," "Aunt/uncle," "Other relative," "Friend or neighbour," "Client(s) of voluntary organisation," and "Other." Anyone selecting "Client(s) of voluntary organisation" was excluded from our sample.

UKHLS' ethnic minority boost was performed via sampling postal sectors with high proportions of ethnic minority groups based on the 2001 census.[2] Invited households were asked a screening question used to select final included participants:[3] *"Does*

anyone living at this address come from or have parents or grandparents from any of the following ethnic groups?"

Outcomes

The SF-36 is extensively validated as a reliable measure across populations,[4] and was adapted to SF-12 for participant ease while maintaining accuracy including for longitudinal research.[5] PCS-12 components relate to physical functioning (limitations in moderate activities/ climbing stairs), physical role limitations (accomplishing less than you would like/ limitations in activities you can do), bodily pain (interfering with normal work), and general health (excellent to poor). MCS-12 components relate to mental health functioning - vitality (energy level), social functioning (health interfering with social activities), emotional role limitations (accomplishing less than you would like/ unable to do activities as carefully), and mental health (feeling calm/ peaceful or downhearted/ blue).[6]

Care characteristics

Residence of recipient (inside or outside household or both) was determined based on the caring questions above. For number of recipients, we calculated a sum of recipients inside and outside household. Weekly hours caring was asked on categorical scale from 0-4 hours to 100+ hours per week/ continuous care. Based on small cell sizes, we combined upper categories (20-34, 35-49, 50-99, 100+ hours) into '20+ hours per week' category. Carers reporting caring inside household were asked which members they cared for, which we used to determine relationship to recipients. For recipients outside household, relationship was asked for the first two. Based on existing literature, three key relationships were considered especially

important: caring for parent, partner, and child. We created three binary variables (given potential for multiple recipients with different relationships) capturing whether participant cared for parent (Y/N), partner (Y/N), child (Y/N), or other (Y/N).

Ethnicity

Ethnicity is captured via the question *"What is your ethnic group?"*, offering single selection among 17 ethnic groups. While we combined several smaller groups into the 'Other' category, we were able to keep the five from the ethnic minority boost separate. The following groups were combined into 'Other:' 'Arab', 'Chinese', 'Gypsy or Irish Traveller', 'Irish', 'Any other Asian background', 'Any other Black background', 'Any other White background', 'White and Asian', 'White and Black African', 'White and Black Caribbean', 'Any other mixed background', and, 'Any other ethnic group'.

Potential confounders

Age, Sex (Male or Female), Marital status (Single/ never married, Married/ civil partnership, Living as a couple, Separated/ divorced, Widowed), Number of children (number of own children in the household under the age of 16 including natural children, adopted children and step children), Highest educational attainment (No qualification, GCSE or equivalent secondary school qualification, A-level or equivalent tertiary school qualification, Other higher education, Degree qualification, or Other qualification), Employment status (Employed (self-employed, paid employment, maternity leave), Unemployed, Retired, Providing family/ home care, Student/ training (full-time student, government training scheme, on apprenticeship), Long-term sick/ disabled, Other (unpaid family business, doing something else)),

Occupational class (National Statistics Socio-Economic Classification (NS-SEC) 3-class version: Management/ professional, Intermediate, Routine, or Not employed), Net monthly equivalised household income (calculated using monthly household income divided by OECD-modified equivalence scale for each household), and baseline Limiting longstanding illness (LLI) (following the approach in existing literature,[7] participants with long-standing illness with difficulties on a limiting area were coded as having LLI; participants without long-standing illness or with long-standing illness but no limiting areas were coded as having no LLI). Two covariates were continuous (age and household income); they were used in continuous form in regression models.

Statistical analysis

Following recommendations in growth curve literature, we applied maximum likelihood estimation, which minimises impact of missing data across waves by using all participants with non-missing outcome in a given wave.[8,9] We included random intercept and random slope to account for the fact that health trajectories may differ at baseline and in rate of change. We used unstructured covariance to allow intercept and slope of health trajectories to correlate. We allowed conditional growth curves by including an interaction term between W1 caring and wave given intercept and slope may vary by W1 caring. We used likelihood ratio tests to assess inclusion of random slopes, addition of quadratic term for wave, and interaction terms between W1 caring and linear and quadratic wave and included items that improved model fit.

Weighting

UKHLS has a complex sample design with multiple boost samples, clustering, and stratification; weights are provided by UKHLS.[10] We used the survey weight at baseline, which takes account of sampling design. We were unable to include weighting for the stratified growth curve models because the models did not converge, possibly as they are not designed for subgroup analysis. We explored this using sensitivity analysis by running unweighted unstratified model to compare vs weighted model to examine magnitude of differences and did not find noteworthy differences (eTable 7).

Results

Mental health trajectory: Pooled (unstratified)

Figure 2 shows pooled conditional growth curves, with carers starting at slightly lower MCS vs non-carers and the two groups converging over time as carers' MCS declines less steeply Waves 2-10. Table of results is shown in eTable 4. After adjusting for covariates, caring is associated with worse mental health at baseline by -1.11 points (-1.33, -0.90). While there is a decline in MCS each wave for all (-0.39; -0.43, -0.35), this decline is reduced among carers vs non-carers (0.09; 0.06, 0.13).

Physical health trajectory: Pooled (unstratified)

Figure 2 also shows pooled conditional growth curves, with carers and non-carers starting at similar PCS and carers declining more steeply Waves 2-10. Table of results is shown in eTable 4. After adjusting for covariates, caring is slightly associated with higher baseline PCS (0.21; -0.03, -0.45). While there is a decline in PCS each wave for all (-0.19; -0.23, -0.15), the decline is steeper among carers vs non-carers (-0.13; -0.17, -0.10).

Comparison of stratified crude model and stratified adjusted model

Given some proposed confounders may mediate the causal pathway for effect modification by ethnicity (e.g. education, employment, occupational class, income), we included the stratified crude model (eFigure 1) in addition to the stratified adjusted model that is shown in the main results (Figure 3). Overall, the shape of results is similar in each ethnic group appear largely similar in the two specifications.

eTable 1. Full sample by wave

	**Sample size**
Wave 1 (Dec 2008 – Mar 2011)	47,015
Wave 2 (Jan 2010 – Mar 2012)	35,946
Wave 3 (Jan 2011 – Jul 2013)	31,388
Wave 4 (Jan 2012 – Jun 2014)	28,721
Wave 5 (Jan 2013 – Jun 2015)	26,619
Wave 6 (Jan 2014 – May 2016)	23,495
Wave 7 (Jan 2015 – May 2017)	22,059
Wave 8 (Jan 2016 – May 2018)	20,533
Wave 9 (Jan 2017 – May 2019)	18,716
Wave 10 (Dec 2017 – May 2020)	17,737

Source: UK Household Longitudinal Study, Waves 1-10.

eTable 2. Comparison of analytical sample with cases excluded due to missingness

	**Analytical sample**		**Excluded due to missingness**		**Total**	**p-value for diff**
	N	% / Mean (SE)	N	% / Mean (SE)	N	
**Caring status**						<0.001
Not carer	39217	83.4%	3558	93.7%	42,775	
Carer	7798	16.6%	240	6.3%	8,038	
Total	47015	100.0%	3798	100.0%	50,813	
**SF-12 PCS**	47,015	49.48 (0.05)	281	51.51 (0.59)	47,296	0.003
**SF-12 MCS**	47,015	50.48 (0.05)	281	50.45 (0.61)	47,296	0.97
**Ethnicity**						<0.001
African	1391	3.0%	143	3.7%	1,534	
Bangladeshi	1104	2.3%	179	4.7%	1,283	
Caribbean	1109	2.4%	119	3.1%	1,228	
Indian	1868	4.0%	202	5.3%	2,070	
Pakistani	1407	3.0%	202	5.3%	1,609	
White	35484	75.5%	2617	68.4%	38,101	
Other	4652	9.9%	364	9.5%	5,016	
Total	47015	100.0%	3826	100.0%	50,841	
**Age (deciles)**						<0.001
16-19	2955	6.3%	432	11.1%	3,387	
20-29	7374	15.7%	774	20.0%	8,148	
30-39	8641	18.4%	664	17.1%	9,305	
40-49	8951	19.0%	736	19.0%	9,687	
50-59	7115	15.1%	549	14.2%	7,664	
60-69	6302	13.4%	364	9.4%	6,666	
70-79	3941	8.4%	214	5.5%	4,155	
80+	1736	3.7%	142	3.7%	1,878	
Total	47015	100.0%	3875	100.0%	50,890	
**Sex**						<0.001
Male	20699	44.0%	2469	63.7%	23,168	
Female	26316	56.0%	1404	36.3%	27,720	
Total	47015	100.0%	3873	100.0%	50,888	
**Marital status**						<0.001
Married/ civil partner	23871	50.8%	2042	53.0%	25,913	

Living as couple	5324	11.3%	398	10.3%	5,722	
Widowed	2832	6.0%	158	4.1%	2,990	
Separated/ divorced	4178	8.9%	106	2.7%	4,284	
Never married	10810	23.0%	1151	29.9%	11,961	
Total	47015	100.0%	3855	100.0%	50,870	
**Number of own children under 16 in household**						0.02
0	33044	70.3%	2796	72.2%	35,840	
1	6188	13.2%	444	11.5%	6,632	
2	5283	11.2%	433	11.2%	5,716	
3+	2500	5.3%	202	5.2%	2,702	
Total	47015	100.0%	3875	100.0%	50,890	
**Highest educational qualification**						<0.001
Degree	10172	21.6%	758	20.0%	10,930	
Other higher education	5256	11.2%	253	6.7%	5,509	
A-level or equivalent	8824	18.8%	753	19.9%	9,577	
GCSE or equivalent	9691	20.6%	811	21.4%	10,502	
Other qualification	4866	10.3%	348	9.2%	5,214	
No qualification	8206	17.5%	861	22.8%	9,067	
Total	47015	100.0%	3784	100.0%	50,799	
**Limiting longstanding illness**						<0.001
No	35460	75.4%	2729	94.9%	38,189	
Yes	11555	24.6%	146	5.1%	11,701	
Total	47015	100.0%	2875	100.0%	49,890	
**Employment status**						<0.001
Employed	25143	53.5%	2288	59.2%	27,431	
Unemployed	3146	6.7%	226	5.9%	3,372	
Retired	9642	20.5%	488	12.6%	10,130	
Family/ home care	3501	7.4%	185	4.8%	3,686	
Student/ training	3463	7.4%	436	11.3%	3,899	
LT sick/ disabled	1789	3.8%	195	5.0%	1,984	
Other	331	0.7%	45	1.2%	376	
Total	47015	100.0%	3863	100.0%	50,878	
**Occupational class**						0.001
Management & professional	10535	22.4%	784	21.3%	11,319	

Intermediate	6137	13.1%	501	13.6%	6,638	
Routine	9300	19.8%	817	22.2%	10,117	
Not employed	21043	44.8%	1575	42.8%	22,618	
Total	47015	100.0%	3677	100.0%	50,692	
**Net equivalized monthly household income (tertiles)**						<0.001
Low	22732	48.4%	1675	43.6%	24,407	
Middle	13726	29.2%	1154	30.1%	14,880	
High	10557	22.5%	1009	26.3%	11,566	
Total	47,015	100.0%	3838	100.0%	50,853	

Source: UK Household Longitudinal Study, Wave 1.
Percentages and sample sizes unweighted

eTable 3. Covariates and caring status by ethnicity

	African (n=1,391)	Bangladeshi (n=1,104)	Caribbean (n=1,109)	Indian (n=1,868)	Pakistani (n=1,407)	White (n=35,484)	Other (n=4,652)	Total (n=47,015)		p-value for % diff
	% / Mean (SE)	% / Mean (SE)	% / Mean (SE)	% / Mean (SE)	% / Mean (SE)	% / Mean (SE)	% / Mean (SE)	% / Mean (SE)	N	
**Age**										<0.001
16-19	9.5%	9.6%	6.1%	6.3%	11.3%	6.4%	5.7%	6.5%	2,955	
20-29	24.8%	31.0%	15.4%	25.7%	29.0%	15.5%	27.0%	16.9%	7,374	
30-39	30.9%	29.7%	16.3%	26.5%	25.6%	14.8%	26.5%	16.4%	8,641	
40-49	22.9%	13.8%	27.3%	17.3%	16.5%	18.2%	18.5%	18.3%	8,951	
50-59	7.2%	7.8%	15.6%	11.9%	9.7%	15.7%	10.0%	15.0%	7,115	
60-69	3.2%	4.4%	7.2%	7.5%	4.6%	14.1%	6.5%	13.0%	6,302	
70-79	1.3%	3.4%	8.3%	3.6%	2.8%	9.4%	4.1%	8.7%	3,941	
80+	0.3%	0.2%	3.7%	1.2%	0.5%	5.8%	1.6%	5.2%	1,736	
Continuous	35.44 (0.38)	35.30 (0.75)	45.05 (0.68)	39.04 (0.54)	35.76 (0.49)	47.67 (0.15)	39.01 (0.29)	46.45 (0.14)	47,015	<0.001
**Sex**										<0.001
Male	47.1%	57.5%	45.5%	54.7%	51.3%	48.8%	47.0%	48.8%	20,699	
Female	52.9%	42.5%	54.5%	45.3%	48.7%	51.2%	53.0%	51.2%	26,316	
**Marital status**										<0.001
Married/ civil partner	43.2%	62.9%	30.9%	63.4%	62.4%	49.5%	46.6%	49.6%	23,871	
Living as couple	6.4%	1.2%	11.2%	1.7%	1.5%	13.0%	13.3%	12.4%	5,324	
Widowed	2.4%	3.0%	4.6%	3.0%	2.5%	7.3%	3.5%	6.7%	2,832	
Separated/ divorced	8.9%	3.6%	11.6%	2.6%	5.6%	7.8%	6.6%	7.6%	4,178	
Never married	39.0%	29.2%	41.7%	29.3%	28.0%	22.4%	30.0%	23.6%	10,810	
**# own children <16 in household**										<0.001
0	53.3%	53.3%	70.1%	65.9%	52.5%	74.5%	67.1%	73.1%	33,044	
1	16.1%	17.2%	15.1%	15.4%	14.6%	11.8%	16.3%	12.4%	6,188	
2	18.3%	13.0%	11.3%	13.6%	15.6%	9.9%	12.0%	10.3%	5,283	
3+	12.3%	16.5%	3.5%	5.1%	17.3%	3.8%	4.6%	4.2%	2,500	
**Highest qualification**										<0.001
Degree	34.0%	22.3%	18.3%	42.4%	27.9%	18.6%	37.0%	20.8%	10,172	
Other higher education	15.2%	5.9%	13.8%	9.9%	7.9%	11.2%	12.0%	11.2%	5,256	

A-level/ equiv	19.9%	22.6%	20.1%	16.8%	17.8%	20.1%	16.7%	19.7%	8,824	
GCSE/ equiv	14.9%	19.0%	22.1%	13.2%	18.1%	22.1%	11.2%	21.0%	9,691	
Other qual	6.3%	7.8%	10.1%	6.2%	8.2%	10.8%	10.3%	10.5%	4,866	
No qual	9.7%	22.4%	15.5%	11.4%	20.2%	17.2%	12.8%	16.7%	8,206	
**LLI**										<0.001
No	89.1%	81.9%	78.9%	84.8%	81.2%	73.8%	83.9%	75.2%	35,460	
Yes	10.9%	18.1%	21.1%	15.2%	18.8%	26.2%	16.1%	24.8%	11,555	
**Employment status**										<0.001
Employed	50.2%	45.0%	51.3%	60.1%	43.6%	55.6%	60.4%	55.8%	25,143	
Unemployed	13.0%	10.4%	13.9%	7.8%	10.2%	5.6%	7.3%	6.0%	3,146	
Retired	3.3%	6.0%	16.8%	8.4%	5.1%	23.9%	9.1%	21.9%	9,642	
Family/ home care	9.5%	18.2%	4.2%	9.2%	21.7%	4.9%	8.1%	5.5%	3,501	
Student/ training	21.1%	17.0%	8.9%	11.6%	14.7%	5.8%	11.8%	6.8%	3,463	
LT sick/ disabled	2.3%	2.8%	4.3%	2.5%	3.5%	3.5%	2.6%	3.4%	1,789	
Other	0.6%	0.6%	0.6%	0.4%	1.2%	0.6%	0.8%	0.7%	331	
**Occupational class**										<0.001
Management/ professional	21.1%	13.2%	21.5%	28.9%	13.6%	23.0%	27.5%	23.3%	10,535	
Intermediate	8.8%	14.2%	12.1%	14.2%	13.2%	13.7%	12.6%	13.6%	6,137	
Routine	21.4%	21.1%	18.4%	20.4%	17.7%	20.8%	22.2%	20.8%	9,300	
Not employed	48.7%	51.6%	48.0%	36.4%	55.5%	42.5%	37.6%	42.3%	21,043	
**Net equivalized monthly household income (tertiles)**										<0.001
Low	61.3%	71.6%	49.1%	42.8%	74.7%	43.7%	45.3%	44.5%	22,732	
Middle	25.2%	19.9%	32.4%	32.9%	18.0%	31.3%	26.6%	30.7%	13,726	
High	13.5%	8.5%	18.4%	24.2%	7.2%	25.0%	28.1%	24.7%	10,557	
Continuous	1126.31 (30.52)	1058.65 (62.64)	1307.41 (42.31)	1512.54 (46.68)	977.08 (39.24)	1528.27 (9.10)	1575.41 (32.09)	1515.65 (8.68)	47,015	<0.001
**Caring status**										<0.001
Carer	5.6%	17.6%	15.6%	11.8%	16.4%	17.3%	9.5%	16.4%	7,798	
Not carer	94.4%	82.4%	84.4%	88.2%	83.6%	82.7%	90.5%	83.6%	39,217	

Source: UK Household Longitudinal Study, Wave 1.
Sample size unweighted, percentage weighted

eTable 4. Multi-level linear regression with SF-12 MCS and SF-12 PCS trajectory waves 1-10

	Model 1		Model 2	
	**Coefficient**	**95% CI**	**Coefficient**	**95% CI**
**SF-12 MCS**				
**Caring status W1**				
Not carer	Ref	-	Ref	-
Carer	-1.07	-1.29- -0.85	-1.11	-1.33- -0.90
**W1 Caring#Wave interaction**				
Not carer#Wave	Ref	-	Ref	-
Carer#Wave	0.09	0.06- 0.13	0.09	0.06- 0.13
**Wave**	-0.38	-0.42- -0.34	-0.39	-0.43- -0.35
**Wave-squared**	0.02	0.01- 0.02	0.02	0.01- 0.02
**Constant**	48.18	47.97- 48.39	49.93	49.50- 50.37
**Random Effects:**				
Var(Wave)	4.52	4.24- 4.81	4.54	4.26- 4.83
Var(Wave-squared)	0.05	0.04- 0.05	0.05	0.04- 0.05
Var(Cons)	49.76	48.38- 51.17	45.18	43.88- 46.51
Cov(Wave, Wave-squared)	-0.43	-0.46- -0.40	-0.43	-0.46- -0.40
Cov(Wave, Cons)	-5.01	-5.52- -4.49	-4.78	-5.29- -4.38
Cov(Wave-squared, Cons)	0.35	0.29- 0.40	0.33	0.28- 0.38
Var(Residual)	40.54	40.00- 41.09	40.50	39.96- 41.05
**SF-12 PCS**				
**Caring status W1**				
Not carer	Ref	-	Ref	-
Carer	0.42	0.16- 0.68	0.21	-0.03- 0.45
**W1 Caring#Wave interaction**				
Not carer#Wave	Ref	-	Ref	-
Carer#Wave	-0.14	-0.17- -0.10	-0.13	-0.17- -0.10
**Wave**	-0.17	-0.21- -0.13	-0.19	-0.23- -0.15
**Wave-squared**	-0.01	-0.01- -0.01	-0.01	-0.01- -0.00
**Constant**	62.45	62.22- 62.67	61.33	60.88- 61.79
**Random Effects:**				
Var(Wave)	4.04	3.81- 4.28	4.05	3.82- 4.30
Var(Wave-squared)	0.04	0.04- 0.04	0.04	0.04- 0.04
Var(Cons)	78.65	76.98- 80.35	59.94	58.52- 61.39
Cov(Wave, Wave-squared)	-0.36	-0.39- -0.34	-0.37	-0.39- -0.34
Cov(Wave, Cons)	-3.52	-3.97- -3.08	-3.25	-3.67- -2.83
Cov(Wave-squared, Cons)	0.15	0.10- 0.20	0.14	0.09- 0.18
Var(Residual)	30.54	30.11- 30.98	30.48	30.05- 30.92

Source: UK Household Longitudinal Study, Waves 1-10.
Weighted using survey weight at baseline.
Model 1 adjusted for baseline age, sex
Model 2 additionally adjusted for baseline marital status, # own children under 16 in household, highest educational qualification, LLI (for MCS), employment status, occupational class, net equivalized monthly household income

eTable 5. Multi-level linear regression with SF-12 MCS and SF-12 PCS trajectory waves 1-10, with ethnicity interaction

	Model 1		Model 2	
	**Coefficient**	**95% CI**	**Coefficient**	**95% CI**
**SF-12 MCS**				
**Caring status W1**				
Not carer	Ref	-	Ref	-
Carer	-0.88	-3.07- 1.32	-0.68	-2.90- 1.55
**W1 Caring#Wave interaction**				
Not carer#Wave	Ref	-	Ref	-
Carer#Wave	-0.28	-0.81- 0.25	-0.29	-0.81- 0.23
**Wave**	-0.34	-0.46- -0.21	-0.34	-0.46- -0.22
**Wave-squared**	0.02	0.01- 0.02	0.02	0.01- 0.02
**Ethnicity#Care#Wave interaction**				
African#Care#Wave	Ref *	-	Ref **	-
Bangladeshi#Care#Wave	0.34	-0.34- 1.02	0.31	-0.36- 0.98
Caribbean#Care#Wave	0.20	-0.42- 0.83	0.23	-0.39- 0.84
Indian#Care#Wave	0.32	-0.27- 0.91	0.34	-0.24- 0.92
Pakistani#Care#Wave	0.43	-0.15- 1.01	0.42	-0.15- 1.00
White#Care#Wave	0.38	-0.15- 0.91	0.39	-0.14- 0.91
Other#Care#Wave	0.36	-0.20- 0.91	0.36	-0.19- 0.91
**Constant**	48.56	48.00- 49.12	50.69	50.00- 51.38
**Random Effects:**				
Var(Wave)	4.52	4.25- 4.82	4.54	4.26- 4.83
Var(Wave-squared)	0.05	0.04- 0.05	0.05	0.04- 0.05
Var(Cons)	49.67	48.29- 51.09	45.11	43.82- 46.45
Cov(Wave, Wave-squared)	-0.43	-0.46- -0.40	-0.43	-0.46- -0.40
Cov(Wave, Cons)	-5.02	-5.53- -4.50	-4.79	-5.29- -4.29
Cov(Wave-squared, Cons)	0.35	0.29- 0.40	0.33	0.28- 0.28
Var(Residual)	40.53	39.99- 41.08	40.49	39.95- 41.04
**SF-12 PCS**				
**Caring status W1**				
Not carer	Ref	-	Ref	-
Carer	-1.59	-3.67- 0.50	-1.83	-3.83- 0.17
**W1 Caring#Wave interaction**				
Not carer#Wave	Ref	-	Ref	-
Carer#Wave	0.17	-0.30- 0.64	0.19	-0.27- 0.65
**Wave**	-0.22	-0.33- -0.11	-0.26	-0.37- -0.15
**Wave-squared**	-0.01	-0.01- -0.01	-0.01	-0.01- -0.00
**Ethnicity#Care#Wave interaction**				
African#Care#Wave	Ref ***	-	Ref ****	-
Bangladeshi#Care#Wave	0.14	-0.42- 0.69	0.11	-0.44- 0.65
Caribbean#Care#Wave	-0.35	-0.92- 0.22	-0.34	-0.90- 0.21
Indian#Care#Wave	-0.14	-0.65- 0.38	-0.14	-0.65- 0.36
Pakistani#Care#Wave	-0.27	-0.80- 0.26	-0.29	-0.81- 0.23
White#Care#Wave	-0.31	-0.78- 0.16	-0.33	-0.79- 0.13
Other#Care#Wave	-0.32	-0.81- 0.17	-0.34	-0.83- 0.14
**Constant**	62.67	62.14- 63.19	62.20	61.55- 62.85
**Random Effects:**				
Var(Wave)	4.04	3.81- 4.29	4.05	3.82- 4.30
Var(Wave-squared)	0.04	0.04- 0.04	0.04	0.04- 0.04
Var(Cons)	78.29	76.61- 80.01	59.71	58.29- 61.17
Cov(Wave, Wave-squared)	-0.37	-0.39- -0.34	-0.37	-0.39- -0.34
Cov(Wave, Cons)	-3.61	-4.06- -3.16	-3.31	-3.74- -2.89
Cov(Wave-squared, Cons)	0.16	0.11- 0.21	0.14	0.10- 0.19
Var(Residual)	30.53	30.10- 30.97	30.47	30.04- 30.90

Source: UK Household Longitudinal Study, Waves 1-10.
Weighted using survey weight at baseline.
* Test for interaction (testparm): F=3.48 p=0.75

** Test for interaction (testparm): F=3.44 p=0.75
*** Test for interaction (testparm): F=13.43 p=0.037
**** Test for interaction (testparm): F=13.32 p=0.038
Model 1 adjusted for baseline age, sex, nativity, ethnicity, ethnicity*care interaction, ethnicity*wave interaction
Model 2 additionally adjusted for baseline marital status, # own children under 16 in household, highest educational qualification, LLI (for MCS), employment status, occupational class, net equivalized monthly household income

eTable 6. Multi-level linear regression with SF-12 PCS trajectory waves 1-10, stratified by ethnic group

	African		Bangladeshi		Caribbean		Indian		Pakistani		White		Other	
	**Coef**	**95% CI**	**Coef**	**95% CI**	**Coef**	**95% CI**	**Coef**	**95% CI**	**Coef**	**95% CI**	**Coef**	**95% CI**	**Coef**	**95% CI**
**Caring status W1**														
Not carer	Ref	-	Ref	-	Ref	-	Ref	-	Ref	-	Ref	-	Ref	-
Carer	-1.93	-3.52- -0.34	-2.01	-3.25- -0.78	-0.43	-1.86-0.99	-1.30	-2.33- -0.27	-1.16	-2.25- -0.08	0.35	0.10-0.60	-0.31	-1.04-0.43
**W1 Caring#Wave interaction**														
Not carer#Wave	Ref	-	Ref	-	Ref	-	Ref	-	Ref	-	Ref	-	Ref	-
Carer#Wave	0.16	-0.22-0.55	0.24	-0.02-0.51	-0.20	-0.47-0.07	0.05	-0.15-0.24	-0.06	-0.27-0.15	-0.14	-0.18- -0.10	-0.17	-0.31- -0.04
**Wave**	-0.64	-0.90- -0.39	-0.96	-1.25- -0.66	-0.26	-0.54-0.02	-0.49	-0.70- -0.29	-0.80	-1.04- -0.56	-0.16	-0.20- -0.12	-0.35	-0.47- -0.23
**Wave-squared**	0.04	0.01-0.07	0.08	0.05-0.12	0.00	-0.03-0.03	0.03	0.01-0.05	0.07	0.04-0.09	-0.01	-0.02- -0.01	0.01	0.00-0.03
**Constant**	63.99	61.61-66.37	65.53	62.19-68.88	62.49	58.82-66.17	61.08	59.08-63.08	61.50	58.82-64.17	62.21	61.62-62.80	60.24	58.93-61.56
**Random Effects:**														
Var(Wave)	2.91	1.80-4.70	1.33	0.37-4.76	4.83	3.43-6.79	3.69	2.76-4.93	2.57	1.52-4.34	3.46	3.27-3.66	3.15	2.59-3.83
Var(Wave-squared)	0.03	0.02-0.05	0.01	0.00-0.07	0.05	0.03-0.07	0.04	0.03-0.05	0.03	0.01-0.05	0.03	0.03-0.03	0.03	0.02-0.04
Var(Cons)	21.81	17.89-26.59	28.71	23.05-35.76	51.09	44.52-58.62	30.43	26.59-34.82	33.21	28.26-39.02	64.53	63.22-65.87	37.78	35.14-40.60
Cov(Wave, Wave-squared)	-0.26	-0.41- -0.11	-0.11	-0.29-0.076	-0.45	-0.63- -0.27	-0.35	-0.46- -0.23	-0.23	-0.38- -0.08	-0.30	-0.32- -0.28	-0.28	-0.35- -0.22
Cov(Wave, Cons)	0.07	-2.01-2.14	0.29	-2.48-3.07	-2.61	-5.40-0.18	-2.24	-3.95- -0.53	-1.02	-3.24-1.19	-2.66	-3.07- -2.25	-1.94	-3.01- -0.87
Cov(Wave-squared, Cons)	-0.12	-0.35-0.11	-0.11	-0.42-0.19	0.13	-0.18-0.44	0.13	-0.05-0.31	-0.06	-0.30-0.18	0.06	0.02-0.11	0.10	-0.02-0.21
Var(Residual)	36.75	34.72-38.90	44.54	41.62-47.67	36.89	34.89-39.01	37.50	35.97-39.09	42.31	40.23-44.51	31.83	31.57-32.08	33.56	32.71-34.44

Source: UK Household Longitudinal Study, Waves 1-10.
Model adjusted for baseline age, sex, marital status, # own children under 16 in household, highest educational qualification, employment status, occupational class, net equivalized monthly household income, and nativity.

eTable 7. Sensitivity analysis: Unweighted multi-level linear regression with SF-12 PCS trajectory waves 1-10

	Model 1		Model 2	
	**Coefficient**	**95% CI**	**Coefficient**	**95% CI**
**SF-12 MCS**				
**Caring status W1**				
Not carer	Ref	-	Ref	-
Carer	-1.07	-1.28- -0.86	-1.12	-1.33- -0.92
**W1 Caring#Wave interaction**				
Not carer#Wave	Ref	-	Ref	-
Carer#Wave	0.09	0.06- 0.13	0.09	0.06- 0.13
**Wave**	-0.35	-0.39- -0.31	-0.36	-0.40- -0.32
**Wave-squared**	0.01	0.01- 0.02	0.01	0.01- 0.02
**Constant**	48.06	47.85- 48.28	49.88	49.45- 50.31
**Random Effects:**				
Var(Wave)	3.69	3.47- 3.92	3.68	3.47- 3.92
Var(Wave-squared)	0.04	0.03- 0.04	0.04	0.03- 0.04
Var(Cons)	49.69	48.60- 50.81	44.49	43.46- 45.54
Cov(Wave, Wave-squared)	-0.34	-0.36- -0.31	-0.34	-0.36- -0.31
Cov(Wave, Cons)	-4.13	-4.55- -3.72	-3.77	-4.17- -3.37
Cov(Wave-squared, Cons)	0.26	0.21- 0.30	0.23	0.19- 0.27
Var(Residual)	43.70	43.38- 44.02	43.71	43.39- 44.03
**SF-12 PCS**				
**Caring status W1**				
Not carer	Ref	-	Ref	-
Carer	0.37	0.13- 0.61	0.15	-0.06- 0.37
**W1 Caring#Wave interaction**				
Not carer#Wave	Ref	-	Ref	-
Carer#Wave	-0.13	-0.16- -0.09	-0.13	-0.16- -0.09
**Wave**	-0.20	-0.23- -0.16	-0.22	-0.26- -0.18
**Wave-squared**	-0.01	-0.01- -0.00	-0.00	-0.01- -0.00
**Constant**	62.38	62.13- 62.64	61.20	60.72- 61.68
**Random Effects:**				
Var(Wave)	3.45	3.27- 3.63	3.44	3.27- 3.62
Var(Wave-squared)	0.03	0.03- 0.03	0.03	0.03- 0.03
Var(Cons)	78.46	77.13- 79.82	58.81	57.72- 59.91
Cov(Wave, Wave-squared)	-0.30	-0.32- -0.28	-0.30	-0.32- -0.28
Cov(Wave, Cons)	-2.69	-3.08- -2.29	-2.33	-2.69- -1.98
Cov(Wave-squared, Cons)	0.06	0.02- 0.11	0.05	0.01- 0.08
Var(Residual)	32.67	32.43- 32.91	32.66	32.43- 32.90

Source: UK Household Longitudinal Study, Waves 1-10.
Model 1 adjusted for baseline age, sex
Model 2 additionally adjusted for baseline marital status, # own children under 16 in household, highest educational qualification, LLI (for MCS), employment status, occupational class, net equivalized monthly household income

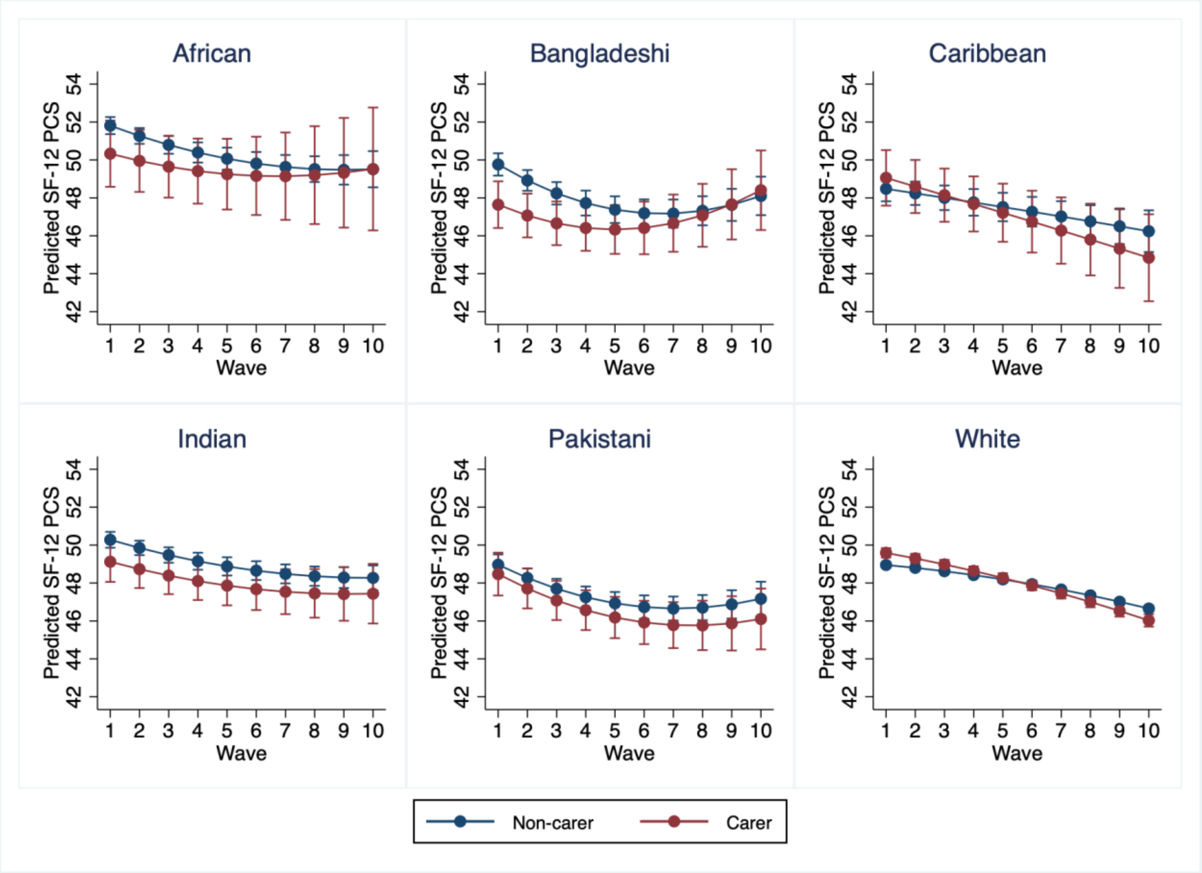

eFigure 1. Predicted SF-12 PCS Waves 1-10 by ethnicity, crude
Source: UK Household Longitudinal Study, Waves 1-10.
Model adjusted for baseline age, sex, and nativity. Model includes linear and quadratic term for wave.

Supplemental References

1. Von Elm E, Altman DG, Egger M, Pocock SJ, Gøtzsche PC, Vandenbroucke JP, et al. The Strengthening the Reporting of Observational Studies in Epidemiology (STROBE) statement: guidelines for reporting observational studies. Annals of internal medicine. 2007;147(8):573–7.

2. McFall S, Nandi A, Platt L. Understanding society: UK household longitudinal study: user guide to ethnicity and immigration research. Institute for Social and Economic Research. 2017;

3. Berthoud R, Fumagalli L, Lynn P, Platt L. Design of the Understanding Society ethnic minority boost sample. Colchester: Institute for Social and Economic Research, University of Essex (Understanding Society Working Paper 2009-02). 2009;

4. McHorney CA, Ware J JE, Lu JF, Sherbourne CD. The MOS 36-Item Short-Form Health Survey (SF-36): III. Tests of Data Quality, Scaling Assumptions, and Reliability across Diverse Patient Groups. Medical care. 1994;32(1):40–66.

5. Jenkinson C, Layte R, Jenkinson D, Lawrence K, Petersen S, Paice C, et al. A shorter form health survey: can the SF-12 replicate results from the SF-36 in longitudinal studies? Journal of Public Health. 1997 Jun 1;19(2):179–86.

6. Ware JE, Kosinski M, Keller SD. SF-12: How to score the SF-12 physical and mental health summery scales. Boston: New England Medical Center; 1998.

7. Booker CL, Sacker A. Limiting long-term illness and subjective well-being in families. LLCS. 2012 Jan 31;3(1).

8. Curran PJ, Obeidat K, Losardo D. Twelve frequently asked questions about growth curve modeling. Journal of cognition and development. 2010;11(2):121–36.

9. Allison PD. Handling missing data by maximum likelihood. In 2012. p. 1038–21.

10. Lynn P. Sample design for understanding society. Underst Soc Work Pap Ser. 2009;2009.
